# Prognostic Value of Cardiac Magnetic Resonance Feature Tracking Strain in Aortic Stenosis

**DOI:** 10.3390/jcdd11010030

**Published:** 2024-01-19

**Authors:** Vasiliki Tsampasian, Ioannis Merinopoulos, Thuwarahan Ravindrarajah, Liam Ring, Ee Ling Heng, Sanjay Prasad, Vassilios S. Vassiliou

**Affiliations:** 1 Department of Cardiology, Norfolk and Norwich University Hospital, Colney Lane, Norwich NR4 7UY, UK; ioannis.merinopoulos@nnuh.nhs.uk (I.M.); thuwa.ravi@googlemail.com (T.R.); 2 Medical School, Faculty of Medicine and Health Sciences, University of East Anglia, Norwich Research Park, Norwich NR4 7UG, UK; 3 Department of Cardiology, West Suffolk Hospital, Hardwick Ln, Bury Saint Edmunds IP33 2QZ, UK; liamring@doctors.org.uk; 4 Royal Brompton Hospital, Royal Brompton and Harefield Hospitals, Guy’s and St Thomas’ NHS Foundation Trust, Sydney Street, London SW3 6NP, UK; eeling.heng@gmail.com; 5 Faculty of Medicine, Imperial College London, London SW7 5NH, UK; s.prasad@rbht.nhs.uk

**Keywords:** aortic stenosis, global longitudinal strain, cardiovascular magnetic resonance, late gadolinium enhancement, ejection fraction

## Abstract

Background: Recent data have suggested that global longitudinal strain (GLS) could be useful for risk stratification of patients with severe aortic stenosis (AS). In this study, we aimed to investigate the prognostic role of GLS in patients with AS and also its incremental value in relation to left ventricular ejection fraction (LVEF) and late gadolinium enhancement (LGE). Methods: We analysed all consecutive patients with AS and LGE-CMR in our institution. Survival data were obtained from office of national statistics, a national body where all deaths in England are registered by law. Death certificates were obtained from the general register office. Results: Some 194 consecutive patients with aortic stenosis were investigated with CMR at baseline and followed up for 7.3 ± 4 years. On multivariate Cox regression analysis, only increasing age remained significant for both all-cause and cardiac mortality, while LGE (any pattern) retained significance for all-cause mortality and had a trend to significance for cardiac mortality. Kaplan–Meier survival analysis demonstrated that patients in the best and middle GLS tertiles had significantly better mortality compared to patients in the worst GLS tertiles. Importantly though, sequential Cox proportional-hazard analysis demonstrated that GLS did not have significant incremental prognostic value for all-cause mortality or cardiac mortality in addition to LVEF and LGE. Conclusions: Our study has demonstrated that age and LGE but not GLS are significant poor prognostic indicators in patients with moderate and severe AS.

## 1. Introduction

Aortic stenosis (AS) is the commonest valvular heart disease in adults, and its prevalence increases exponentially with age [1]. The complex pathophysiology of aortic stenosis is reflected in its impact on the left ventricular (LV) haemodynamics and remodelling [2]. The subsequent LV pressure overload results in increased wall thickness and hypertrophy, which has been proved to be maladaptive and is associated with increased morbidity and mortality [3,4]. Over the last few years, research efforts have focused on imaging subclinical changes in LV function and structure. Cardiovascular magnetic resonance (CMR), with its ability to provide information about anatomy, function and tissue characterisation, has helped immensely improve our understanding of LV changes [5]. It has been shown that midwall fibrosis, as assessed by late gadolinium enhancement (LGE), is an independent predictor of mortality in AS, out to 5 years of follow up, offering incremental prognostic value to left ventricular ejection fraction (LVEF) [4,5,6,7]. Even though gadolinium administration is overall safe, the number of associated concerns, including nephrogenic systemic fibrosis and possible accumulation in the brain and cost, encourage the search for alternative biomarkers [8].

Global longitudinal strain (GLS) has been suggested as a marker of subclinical LV decompensation. It is affected by the development of fibrosis in patients with AS and the pattern of LV remodelling with worse values in cases of severe concentric left ventricular hypertrophy [9,10]. In addition, some recent data have shown that GLS could be useful for risk stratification of asymptomatic patients with severe AS and normal LVEF and strain in general can aid management in other valvulopathies also [11,12]. 

In this study, we aimed to investigate the prognostic role of GLS in patients with aortic stenosis and also its incremental prognostic value in relation to LVEF and LGE.

## 2. Materials and Methods

This prospective study (ClinicalTirals.gov Identifier: NCT00930735) [13] included all consecutive patients with AS and late gadolinium contrast-enhanced CMR (LGE-CMR) from the Royal Brompton Hospital, London. The most recent AHA/ACC guidelines for management of patients with valvular heart disease were used to assess AS severity in all patients, even if recruited prior to this [14]. Patients with acute coronary syndrome, clinical suspicion or evidence of infection, disseminated malignancy, severe aortic regurgitation, more than moderate mitral regurgitation or stenosis, previous valve replacement, contraindication to CMR (including the presence of nonconditional devices) and estimated glomerular filtration rate of <30 mL/min were excluded. 

This study was approved by the NHS England Research Ethics Committee (reference 07/H0708/83) and was undertaken according to the ethical standards of the Declaration of Helsinki. Written informed consent was provided by all patients.

### 2.1. Cardiovascular Magnetic Resonance

CMR scans were undertaken on a 1.5T scanner (Magneton Sonata or Avanto, Siemens, Erlangen, Germany) according to a standardised protocol described previously [13]. Briefly, following initial localiser images, vertical long-axis (VLA) cine with balanced steady-state free precession (SSFP) at end-expiration were acquired. These guided SSFP cine were in the two-, three- and four-chamber views. Contiguous 10 mm short-axis slices of the LV were then taken from base to apex. Cine acquisition was undertaken preferably with retrospective ECG gating. In the case of arrhythmia, prospective triggering was used. Aortic valve planimetry and LV mass and volume were subsequently calculated. Following gadolinium (Gadovist, Schering AG, Berlin, Germany) infusion, inversion recovery-prepared spoiled gradient-echo images were acquired in standard long- and short-axis views to detect areas of LGE [15]. 

Analysis of images was undertaken offline on dedicated software for LV function, volumes, mass and AS severity (CMR Tools, Cardiovascular Imaging Solutions., London, United Kingdom). Manual contour selection was performed in end-systole and end-diastole on the epicardial and endocardial walls in two-, three- and four-chamber long-axis, and all short-axis cine slices to enable LVEF calculation. LV myocardial fibrosis was quantified using FWHM via a separate dedicated software (CVI42, Circle Cardiovascular Imaging, Calgary, AB, Canada). Strain was expressed as the percentage change in length of a small element of the myocardium in relation to its original length. This was measured along the radial, circumferential and longitudinal cardiac axes. Strain analysis was performed on patients using an MR-FT software package (CVI42, Tissue tracking module, Circle Cardiovascular Imaging, Calgary, AB, Canada). End-diastolic and end-systolic LV contours were manually traced on the epicardial and endocardial walls in two-, three- and four-chamber long-axis and all short-axis cine slices. After manually defining the RV insertion points, an automated feature-tracking strain analysis produced 3D strain calculations, which provided data for global longitudinal, circumferential and radial strain. 

The American Heart Association (AHA) 16-segment model was used to obtain the individual segment strain values for each axis. The images were analysed by two independent, blinded observers and reviewed by an experienced level 3 SCMR/EACVI operator with more than 10 years’ experience in CMR. Excellent inter- and intra-observer reproducibility as well as inter-study repeatability of this software have already been demonstrated in recent studies [16,17].

### 2.2. Statistical Analysis

Survival data were obtained from the office of national statistics, a national body where all deaths in England are registered by law. Death certificates were obtained from the general register office and adjudicated by an independent committee. Baseline data are presented as mean ± SD for continuous variables and number (proportion) for categorical variables. Univariate and multivariate Cox regression analyses were undertaken to identify possible predictors of all-cause and cardiac mortality. Kaplan–Meier estimator plots were used to plot survival.

## 3. Results

Patient demographics are shown in Table 1. Some 194 consecutive patients with aortic stenosis were investigated with CMR at baseline and followed up for 7.3 ± 4 years. The majority of patients (141, 72.7%) had severe aortic stenosis.

There were 86 deaths overall, and 40 of these deaths were classified as cardiac by an independent adjudicating committee. Univariate Cox regression analysis identified increasing age, chronic kidney disease (CKD), previous percutaneous coronary intervention (PCI), LGE (any pattern), indexed LA volume, gadolinium mass, GLS, LVEF, RVEF, indexed LVESV and use of aldosterone antagonist as poor prognostic factors for all-cause and cardiac mortality (Table 2). Hypertension and diuretic use were identified as poor prognostic factors for all-cause mortality but not for cardiac mortality. Aortic valve intervention (SAVR or TAVR) was associated with a better prognosis for both all-cause and cardiac mortality.

However, on multivariate analysis, only increasing age remained significant for both all-cause and cardiac mortality, while LGE (any pattern) retained significance for all-cause mortality and had a trend to significance for cardiac mortality, indicating that our study was not sufficiently powered to detect an effect as the HR was high (Table 3).

**Figure 1:**
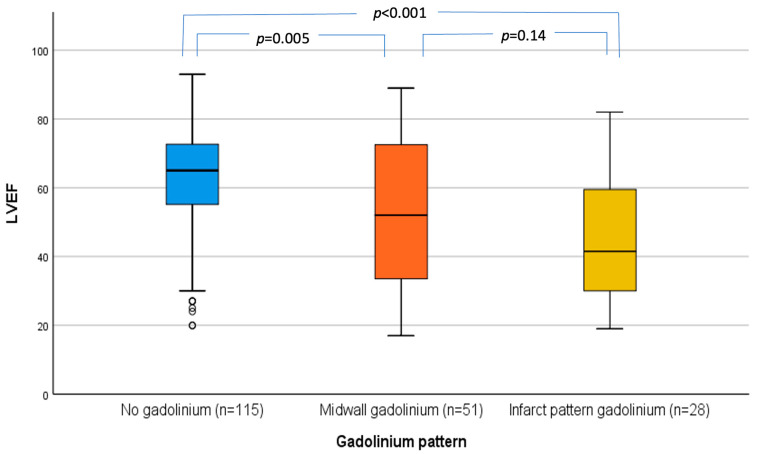
Box and whisker plots showing the distribution of LVEF by pattern of LGE on CMR. Outlier data are included in the plot. CMR: cardiac magnetic resonance, LGE: late gadolinium enhancement, LVEF: left ventricle ejection fraction.

As shown in Figure 1, there was a relationship between LGE and LVEF, with progressively decreasing LVEF as moving from no LGE to midwall LGE to infarct-pattern LGE. Patients without LGE had significantly higher LVEF compared to patients with midwall LGE (61 ± 16 vs. 52 ± 21; *p* = 0.005) or infarct-pattern LGE (61 ± 16 vs. 45 ± 18; *p* < 0.001). There was no difference in the LVEF between patients with midwall LGE and patients with infarct-pattern LGE (52 ± 20 vs. 45 ± 18; *p* = 0.14).

**Figure 2:**
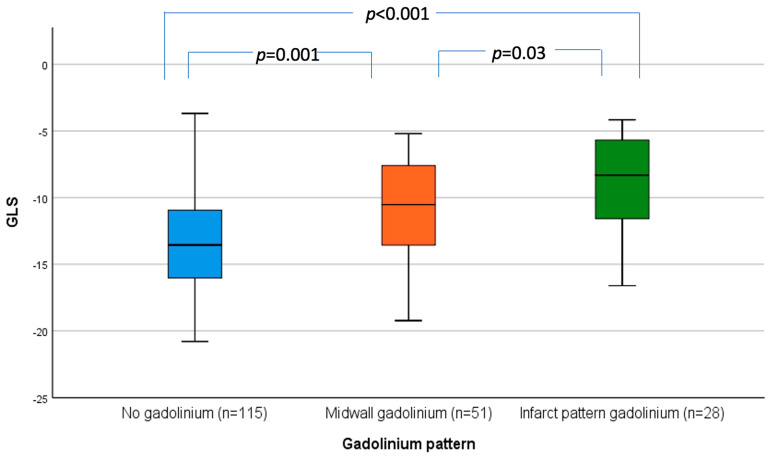
Box and whisker plot showing the distribution of GLS by pattern of LGE on CMR. CMR: cardiac magnetic resonance, GLS: global longitudinal strain, LGE: late gadolinium enhancement.

Similarly, GLS progressively became less negative (i.e., got worse) when moving from no LGE to midwall LGE to infarct-pattern LGE (Figure 2). Patients without LGE had significantly better GLS compared to patients with midwall LGE (−13.3 ± 3.7 vs. −11.1 ± 3.9; *p* = 0.001) or infarct-pattern LGE (−13.3 ± 3.7 vs. −9.1 ± 3.9; *p* < 0.001). Patients with midwall LGE also had significantly less GLS compared to patients with infarct-pattern LGE (−11.1 ± 3.9 vs. −9.1 ± 3.9; *p* = 0.035).

**Figure 3:**
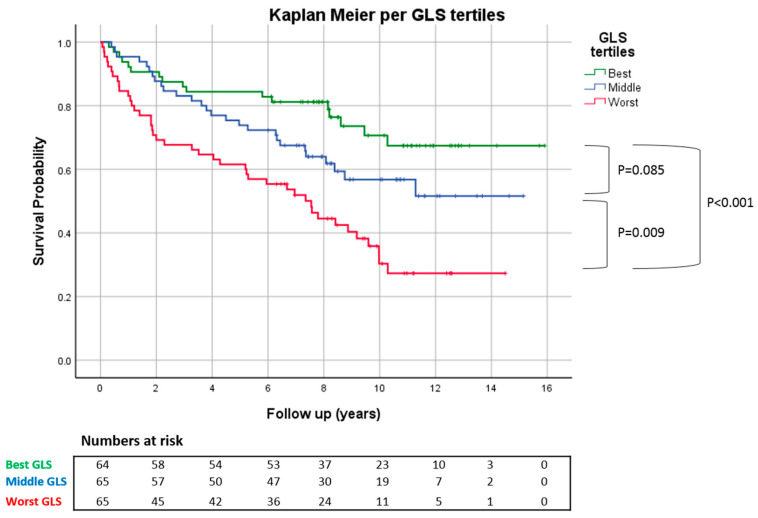
Kaplan–Meier survival plot showing that patients in top two GLS tertiles had significantly better survival compared to bottom tertile, while there was no significant difference between the top two GLS tertiles. GLS: global longitudinal strain.

Kaplan–Meier survival analysis demonstrated that patients in the best and middle GLS tertiles had significantly better mortality compared to patients in the worst GLS tertiles, whilst there was no significant difference between the best and middle GLS tertiles (Figure 3).

**Figure 4:**
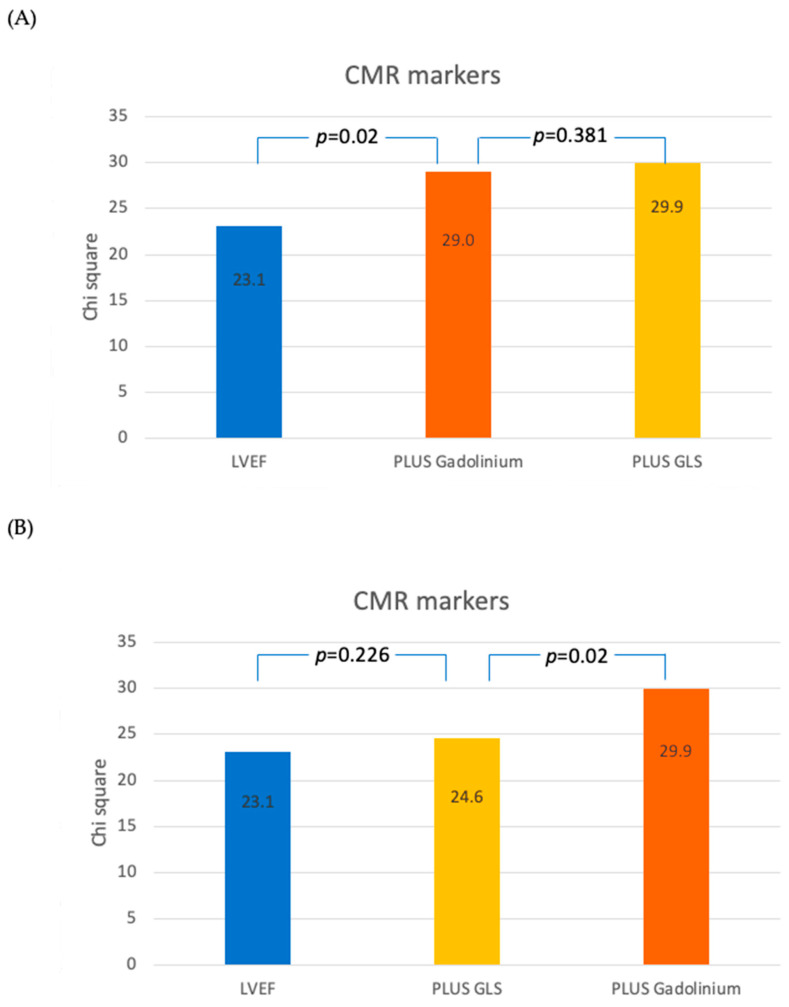
Sequential Cox proportional-hazard models demonstrating the incremental discriminatory value of differing CMR markers for all-cause mortality. (**A**) EF -> Gadolinium -> GLS; (**B**) EF -> GLS -> Gadolinium.

**Figure 5:**
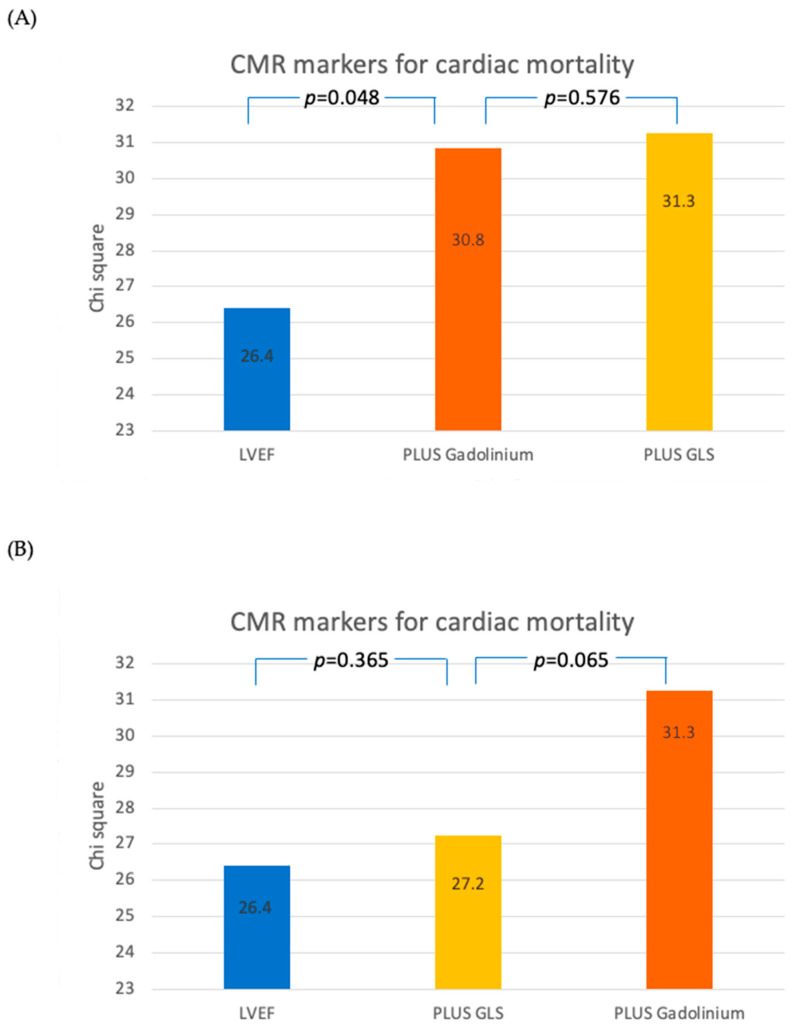
Sequential Cox proportional-hazard models demonstrating the incremental discriminatory value of differing CMR markers for cardiac mortality. (**A**) EF -> Gadolinium -> GLS; (**B**) EF -> GLS -> Gadolinium.

Importantly though, sequential Cox proportional-hazard analysis demonstrated that GLS did not have significant incremental prognostic value for all-cause mortality (Figure 4A,B) or cardiac mortality (Figure 5A,B), in addition to LVEF and LGE, whilst the presence of midwall fibrosis did.

## 4. Discussion

Aortic stenosis is one of the most important and commonest causes of valvular heart disease [18]. The increasingly ageing population and the development of percutaneous therapeutic options for traditionally nonsurgical candidates make even more relevant than previously the need for a comprehensive preoperative assessment and risk stratification [19]. It is well recognised that LGE has an incremental prognostic value to LVEF [20]. However, certain concerns, such as nephrogenic systemic fibrosis and possible gadolinium accumulation in the brain, mean that gadolinium administration might not be possible to all patients. It is now widely accepted that strain imaging reflects better the LV systolic function and, for this purpose, GLS has become a robust and easily accessible method in clinical practice [21]. Additionally, CMR-derived myocardial strain has been found to be an alternative means of assessment of the LV function, similar to speckle-tracking echocardiography [22]. 

In this study, we investigated whether CMR-derived GLS is an independent prognostic factor in patients with AS and whether it carries significant incremental prognostic value when added to parameters that are routinely used in clinical practice, such as LVEF and LGE. We have demonstrated that GLS is an adverse prognostic factor for all-cause and cardiac mortality on univariable analysis but loses its significance in multivariable analysis and has no significant incremental prognostic value to LVEF and LGE.

It has been shown that early LV dysfunction can be detected by changes in the GLS, while other conventional parameters such as the EF fail to do so [23,24,25]. A recent meta-analysis of echocardiographic individual participant data demonstrated that, in patients with asymptomatic, severe AS and LVEF > 50%, impaired GLS on echocardiography (<14.7%) was a strong independent predictor of mortality in multivariable analysis including age, gender, indexed aortic valve area and LVEF [11]. It suggested that GLS is better than LVEF to detect subclinical LV dysfunction and guide the management of asymptomatic patients with severe AS and preserved LV function. The prognostic significance of GLS is supported by large registry data as well, which have furthermore shown that patients with preserved LVEF but impaired GLS (worse than −14%) have poor prognosis similar to patients with impaired LVEF before aortic valve intervention [26]. It has also been shown previously that 2D longitudinal strain, in patients with severe AS undergoing TAVI, was able to predict recovery of myocardial function post TAVI and that its change is closely related to symptomatic improvement [25]. However, none of the echocardiographic parameters studied including longitudinal strain were able to predict postoperative major adverse cardiac events or 30-day mortality [25]. Improvement of the GLS after TAVI has also been associated with better prognosis and lower mortality [27,28]. 

This study demonstrated that CMR-derived GLS is strongly associated with mortality, with patients in the better GLS tertiles having significantly better survival rates compared to the ones in the lower GLS tertiles. This finding is in keeping with other studies that showed a prognostic significance of both echocardiography- and CMR-derived GLS in patients with aortic stenosis as well as other cardiac diseases, such as myocardial infarction and heart failure with preserved ejection fraction [29,30]. The association of GLS and survival may be explained by the recent evidence demonstrating its correlation with irreversible replacement fibrosis in patients with aortic stenosis. In a study that included 261 patients with moderate and severe aortic stenosis, Le et al., demonstrated that echocardiographic GLS has high sensitivity and specificity (95% for each) to detect the presence of replacement fibrosis [31]. Replacement fibrosis is an important independent prognostic marker associated with poor long-term outcomes in patients with aortic stenosis [32]; therefore, its detection has important clinical implications for the patient.

Importantly, however, our study also demonstrated that CMR-derived GLS did not have significant incremental prognostic value when added to the conventionally used CMR parameters, including LVEF and LGE. This is in contrast with previous echocardiographic studies that have found an important additive value of GLS on the LVEF [33,34]. This discrepancy may be explained by the differences in the methodology of volumetric assessment between the two imaging modalities. The LVEF assessment and calculation by echocardiography is based on several geometric assumptions and is less reproducible than the CMR volumetric assessment [35,36]. These limitations in echocardiography are often overcome by strain imaging, which arguably provides a better assessment of the LV function with the ability to detect subtle subclinical changes even when the echocardiographic LVEF is within normal range [37]. Thus, the majority of echocardiographic studies performed so far are in agreement that GLS has an important additive value to the LVEF.

With CMR being the gold standard in volumetric assessment and tissue characterisation, it is important to understand how CMR-derived GLS may add to the prognostic strength of routinely used parameters. While there are studies examining the role of the CMR-derived GLS on long-term outcomes, there are limited and conflicting data evaluating its incremental value when added to the LVEF and LGE presence obtained by CMR [29,30,38,39]. In a study of 323 patients following ST-elevation myocardial infarction, Schuster et al. demonstrated that GLS was significantly associated with major adverse cardiovascular events [39]. However, when added to the traditional CMR parameters, GLS did not significantly improve risk reclassification. On the other hand, a study of 210 patients with dilated cardiomyopathy (DCM) showed that GLS was an independent predictor of survival and offered significant incremental value in the risk stratification of patients with DCM [40]. 

Our study is the first large study to examine the additive value of GLS in patients with AS. Patients with AS may exhibit diffuse myocardial fibrosis as well as replacement (focal) myocardial fibrosis, with only the latter being expressed as LGE [32,41]. While the association between GLS and replacement fibrosis (LGE) has previously been shown, it is unclear how well GLS correlates with diffuse fibrosis. To make matters more complicated, the degree of the two types of fibrosis in patients with AS may vary significantly among patients, which may make the additive prognostic value of GLS even more challenging in this population. In this study, we have demonstrated that, although GLS is associated with long-term survival, when added to the LVEF and the LGE, it does not have significant incremental prognostic value. This observation does not undermine the prognostic value of GLS on survival but it may highlight the robustness of the currently used parameters, such as the LGE. As such, GLS can still be of significant value in addition to LVEF when administration of gadolinium is not possible due to contraindications, patient preference or financial reasons. It is clear, though, that there is a crucial need for further studies in this field to assess the additive value of CMR-derived GLS on the conventionally used parameters across different cardiac diseases and its correlation with the traditionally used parameters. 

Another interesting finding of our study is that the AS severity was not found to be a significant prognostic factor in the univariate analysis. However, this finding may not be entirely surprising, when valve intervention is taken into consideration. In the 7.3 ± 4 years of follow-up, 89% of the patients with severe AS and 58% of the patients with moderate AS had valve intervention (TAVR or SAVR), indicating that, during the follow-up period, many “moderate AS” progressed to “severe AS”. It is therefore very likely that the prognosis of the patients was not adversely affected because the majority of the patients had valve intervention at an appropriate timing. Consequently, the severity of the AS at baseline would not be a prognostic factor as the valve intervention has acted favourably in terms of long-term prognosis. On the other hand, conditions that could not be reversed by the intervention (such as age and replacement myocardial fibrosis) were found to be significant prognostic factors for mortality. Furthermore, it is noteworthy that recent evidence has shown that moderate AS may not be as a benign condition as previously thought. Patients with moderate AS have been found to have poor long-term outcomes, with their 5-year mortality risk being similar to that of the patients with severe AS [42,43]. This evidence highlights that the prognostic trajectory of patients with moderate AS may be analogous to that of the patients with severe AS.

## 5. Limitations

Whilst our cohort is large, it is still possible that we were underpowered to detect a smaller adverse effect associated with GLS, even when LGE had been used. However, in line with other studies, we have demonstrated that age is a significant poor prognostic indicator for all-cause and cardiac mortality and that LGE is a significant poor prognostic indicator for all-cause mortality, indicating that our sample size was decent. LGE did not reach statistical significance for cardiac mortality, which is probably related to the size of our cohort.

## 6. Conclusions

Our study has demonstrated that age and LGE but not GLS are significant poor prognostic indicators in patients with moderate and severe AS. We have demonstrated that GLS can be easily undertaken via postprocessing in patients with aortic stenosis. However, we failed to observe an added benefit for prognostication in our patients, unlike what is seen with LGE. We conclude that, whilst GLS can be calculated easily from CMR for patients with aortic stenosis, at present, we are not able to associate it significantly with prognosis or derive a cut-off for clinical use, and a lower LVEF and presence of any fibrosis at LGE imaging remain the two stronger adverse predictors of events in patients with aortic stenosis. It may have a role, however, in patients where administration of gadolinium is not possible and further studies can be utilised to evaluate this.

## Figures and Tables

**Table 1:** Baseline patient data.

Demographics	Whole Cohort (N = 194)	Moderate AS (N = 53)	Severe AS (N = 141)
Age, years	72.9 ± 12.9	68.1 ± 14.7	74.7 ± 11.8
Male, n (%)	118 (60.8)	38 (71.7)	80 (56.7%)
Hypertension, n (%)	87 (44.8)	30 (56.6)	57 (40.4)
Diabetes, n (%)	43 (22.2)	9 (17)	34 (24.1)
Hypercholesterolaemia, n (%)	96 (49.5)	24 (45.3)	72 (51.1%)
Chronic kidney disease, n (%)	33 (17)	8 (15.1)	25 (17.7)
Atrial fibrillation, n (%)	41 (21.1)	8 (15.1)	33 (23.4)
Previous coronary artery bypass, n (%)	22 (11.3)	5 (9.4)	17 (12.1)
Previous percutaneous coronary intervention, n (%)	27 (13.9)	4 (7.5)	23 (16.3)
Pharmacotherapy			
ACE-I/ARB, n (%)	91 (46.9)	29 (54.7)	62 (44)
Betablocker, n (%)	67 (34.5)	23 (43.4)	44 (31.2)
Aldosterone antagonist, n (%)	30 (15.5)	10 (18.9)	20 (14.2)
Statins, n (%)	121 (62.4)	34 (64.2)	87 (61.7)
Diuretic, n (%)	71 (36.6)	12 (22.6)	59 (41.8)
Warfarin, n (%)	27 (13.9)	11 (20.8)	16 (11.3)
CMR data			
CMR aortic valve area, cm^2^	0.89 ± 0.28	1.25 ± 0.24	0.76 ± 0.16
LVEF, %	57 ± 19	58 ± 20	56±18
LV mass, g	143 ± 73	180 ± 66	128 ± 69
Indexed LV mass	75.9 ± 34.7	92.7 ± 28.5	69.7 ± 34.8
No myocardial fibrosis, n (%)	115 (59.3)	21 (39.6)	94 (66.7)
Midwall fibrosis, n (%)	51 (26.3)	22 (41.5)	29 (20.6)
Infarction pattern fibrosis, n (%)	28 (14.4)	10 (18.9)	18 (12.8)
LGE mass	4.7 ± 8.5	6.4 ± 8.7	4 ± 8.4
LGE, %	2.9 ± 6.6	3.4 ± 5.1	2.7 ± 7.1
GLS	−12.2 ± 4.1	−12.3 ± 4.3	−12.1 ± 4.1
RVEF, %	59 ± 11.8	56 ± 11.2	60 ± 11.7
Indexed LA volume	60.6 ± 22.9	65.3 ± 25.4	58.8 ± 21.7
Indexed LVEDV	127.9 ± 61.2	106.3 ± 49.8	136.0 ± 63.3
Indexed LVESV	48.0 ± 35.3	48.0 ± 38.7	48.4 ± 34.1
Intervention			
None, n (%)	38 (19.6)	22 (41.5)	16 (11.3)
TAVR, n (%)	92 (47.4)	4 (7.5)	88 (62.4)
SAVR, n (%)	64 (33)	27 (50.9)	37 (26.2)

Values are mean ± SD unless stated otherwise. ACE-I: angiotensin converting enzyme inhibitor, ARB: angiotensin II receptor blocker, CMR: cardiovascular magnetic resonance, GCS: global circumferential strain, GLS: global longitudinal strain, GRS: global radial strain, LA: left atrium, LGE: late gadolinium enhancement, LVEF: left ventricular ejection fraction, RVEF: right ventricular ejection fraction, SAVR: surgical aortic valve replacement, TAVR: transcatheter aortic valve replacement.

**Table 2:** Univariate Cox regression analysis for all-cause and cardiac mortality.

Variable	All-Cause Mortality		Cardiac Mortality	
	*p* Value	HR [95% CI]	*p* Value	HR [95% CI]
Male	0.241	0.773 [0.502, 1.189]	0.425	0.744 [0.413, 1.452]
Age	<0.001 *	1.044 [1.022, 1.066]	0.009 *	1.042 [1.010, 1.074]
Hypertension	0.011 *	1.747 [1.134, 2.692]	0.150	1.600 [0.844, 3.034]
Diabetes	0.473	1.199 [0.731, 1.965]	0.483	1.295 [0.629, 2.667]
Hypercholesterolaemia	0.953	1.013 [0.660, 1.554]	0.825	0.931 [0.492, 1.760]
Chronic kidney disease	0.003 *	2.097 [1.278, 3.440]	0.003 *	2.867 [1.445, 5.689]
Atrial fibrillation	0.526	1.177 [0.711, 1.947]	0.914	0.958 [0.440, 2.086]
Previous CABG	0.477	1.249 [0.677, 2.301]	0.433	1.416 [0.593, 3.378]
Previous PCI	0.018 *	1.933 [1.122, 3.330]	0.029 *	2.290 [1.088, 4.820]
Gadolinium (midwall or infarct pattern)	0.001 *	2.138 [1.391, 3.286]	0.001 *	2.890 [1.507, 5.541]
Gadolinium mass	0.024 *	1.024 [1.003, 1.045]	0.042 *	1.030 [1.001, 1.060]
Gadolinium %	0.134	6.710 [0.557, 80.858]	0.168	9.740 [0.384, 247.061]
Indexed LV mass	0.429	1.002 [0.996, 1.009]	0.252	1.005 [0.996, 1.014]
Indexed LA volume	0.015 *	1.010 [1.002, 1.018]	0.047 *	1.011 [1.000, 1.023]
LVEF	<0.001 *	0.974 [0.963, 0.985]	<0.001 *	0.960 [0.944, 0.976]
Indexed LVEDV	0.822	1.000 [0.997, 1.004]	0.785	1.001 [0.996, 1.006]
Indexed LVESV	0.008 *	1.007 [1.002, 1.013]	0.001 *	1.013 [1.006, 1.020]
RVEF	<0.001 *	0.968 [0.951, 0.985]	0.002 *	0.962 [0.938, 0.986]
GLS	<0.001 *	1.126 [1.067, 1.188]	<0.001 *	1.201 [1.104, 1.306]
GRS	<0.001 *	0.970 [0.955, 0.985]	<0.001 *	0.951 [0.926, 0.976]
GCS	<0.001*	1.120 [1.070, 1.173]	<0.001 *	1.170 [1.090, 1.256]
CMR AS severity	0.351	0.809 [0.518, 1.263]	0.261	0.692 [0.364, 1.316]
ACE-I/ARB	0.546	1.143 [0.741, 1.763]	0.524	0.811 [0.426, 1.545]
Betablocker	0.824	1.052 [0.672, 1.647]	0.723	1.124 [0.589, 2.143]
Statin	0.194	1.365 [0.854, 2.182]	0.775	0.910 [0.477, 1.736]
Aldosterone antagonist	0.018 *	1.879 [1.114, 3.169]	0.001 *	3.252 [1.669, 6.336]
Diuretic	0.002 *	2.114 [1.329, 3.363]	0.084	1.798 [0.923, 3.499]
Aortic valve intervention (SAVR or TAVR)	<0.001 *	0.329 [0.210, 0.516]	<0.001 *	0.174 [0.093, 0.325]

* Indicates significance at the <0.05 level. AS: aortic stenosis, ACE-I: angiotensin converting enzyme inhibitor, ARB: angiotensin II receptor blocker, CABG: coronary artery bypass graft, CMR: cardiovascular magnetic resonance, GCS: global circumferential strain, GLS: global longitudinal strain, GRS: global radial strain, LA: left atrium, LVEF: left ventricular ejection fraction, RVEF: right ventricular ejection fraction, SAVR: surgical aortic valve replacement, TAVR: transcatheter aortic valve replacement.

**Table 3:** Multivariate Cox regression models showing age as a significant poor predictor of all-cause and cardiac mortality, while gadolinium remained significant only for all-cause mortality.

Variable	All-Cause Mortality		Cardiac Mortality	
	*p* Value	HR [95% CI]	*p* Value	HR [95% CI]
Age	<0.001 *	1.044 [1.021, 1.067]	0.020 *	1.041 [1.006, 1.078]
Gadolinium (midwall or infarct pattern)	0.018 *	1.752 [1.100, 2.790]	0.080	1.899 [0.927, 3.892]
Chronic kidney disease	0.130	1.486 [0.889, 2.484]	0.113	1.778 [0.872, 3.624]
Indexed LA volume	0.760	1.002 [0.992, 1.012]	0.891	0.999 [0.984, 1.014]
LVEF	0.353	0.991 [0.972, 1.010]	0.119	0.977 [0.949, 1.006]
GLS	0.376	1.040 [0.953, 1.135]	0.587	1.039 [0.905, 1.192]

* Indicates significance at the <0.05 level. LA: left atrium, LVEF: left ventricular ejection fraction, GLS: global longitudinal strain.

## Data Availability

The data presented in this study are available on request from the corresponding author. The data are not publicly available due to ethical restrictions.
